# Svante Pääbo, reader of the Neanderthal genome

**DOI:** 10.1111/apha.13902

**Published:** 2022-11-23

**Authors:** Karolina Wielgus, Mikołaj Danielewski, Jarosław Walkowiak

**Affiliations:** ^1^ Department of Pediatric Gastroenterology and Metabolic Diseases Poznan University of Medical Sciences Poznań Poland

Svante Pääbo is a pioneer in paleogenetics who received this year's Nobel Prize in Physiology or Medicine for his scrutiny of genetic material from ancient samples, including long‐extinct species.

His first scientific breakthrough came in 1985, less than a year after Russel Higuchi isolated and sequenced short fragments of mitochondrial DNA from a 140‐year‐old museum specimen of an extinct animal. Svante Pääbo tested 23 different Egyptian mummies for the presence of genetic material and obtained nuclear DNA from a 2400‐years‐old mummy of a 1‐year‐old boy. Most DNA fragments were below 500 bp (*base pair*) long, but a portion was around five kbp long. DNA was amplified through molecular cloning, which gave, among others, a product with an insert of 3.4 kbp. Southern hybridization showed that the insert contained two sets of *Alu* repeats, transposable elements used as genetic markers in the genomes of primates. Sequencing revealed one fragment, with 500 bp of flanking DNA, which is 77% homologous with the human *Alu* consensus sequence. Only 30% of all point mutations were transversions. These results are consistent with divergence patterns in *Alu* repeats known thus far. Therefore, no significant post‐mortem damage occurred.^1^ It was an impressive achievement for several reasons: Firstly, the sampled material was ancient. Secondly, the obtained DNA was nuclear, which, unlike mitochondrial DNA, does not exist in cells in many copies. Lastly, Pääbo and his team achieved this with molecular cloning, as PCR was not yet available. Together, Higuchi's and Pääbo's opened the door to ancient genomics through which the scientific community treads.

Curiosity‐driven, Pääbo and his team extracted DNA from a Neanderthal‐type specimen found in Germany in 1856, while working at the Zoological Institute of the University in Munich. This was in 1997. Sequencing brought to light a previously unknown mitochondrial DNA sequence, which is a hypervariable part of the mitochondrial control region. Extensive testing, including an independent extraction in a different laboratory, confirmed that the sequence was endogenous and that nucleotide misincorporation had not influenced the outcome. Thus, Neanderthal mtDNA diverged from modern human mtDNA 550 000 to 690 000 years ago, long before the arrival of the most recent common ancestor for modern human mtDNA. As the sequence in question diverged from the common genetic line between Humans and Neanderthals, the Neanderthals did not contribute to the mitochondrial genome of today's humans.^2^

Our mitochondrial DNA separated from the Neanderthal around 660 000 years ago, as shown in 2008 when Pääbo's team sequenced the complete Neanderthal mitochondrial genome from a bone of a 38 000‐year‐old individual. What is more, Neanderthals have a significantly greater proportion of nonsynonymous to synonymous mutations in their sequences of the protein‐coding mitochondrial genes. Perhaps this is a sign of lower purifying pressure resulting from a smaller population size. Moreover, the Neanderthal mitochondrial lineage was 20% shorter than a human one, thrice what would be expected from a side‐effect due to the sample age. This difference was attributed to random chance affecting the amounts of substitutions.^3^

In 2010, the first draft of a Neanderthal nuclear genome was sequenced, using the samples from Vindija Cave in Croatia, altogether 21 ancient bones. It was achieved with highly robust scientific methods and a great dose of adaptability. For example, high contamination of endogenous genetic material with exogenous one is a common challenge. Therefore, Pääbo's team decided to use restriction enzymes specific to bacterial DNA, which resulted in a 4‐ to 6‐fold increase in the proportion of Neanderthal DNA in libraries. The analysis of the reconstructed genome revealed that relatively few amino acid changes became fixed in the human genome in the last couple of hundred thousand years. The most affected genes were: *SPAG17*, a coding protein crucial for the sperm flagellum, *PCD16* coding protein potentially involved in wound healing, *TTF1* which regulates ribosomal gene transcription, *CAN15* which function is unknown, and RPTN expressed in the epidermis. The results also showed that the Neanderthals were more closely related to non‐Africans than Africans and confirmed the gene flow from Neanderthals to anatomically modern humans. Since Europeans and Asians showed to be more closely related, it was theorized that the gene flow occurred before these two groups diverged from one another. The average divergence of anatomically modern humans and Neanderthal DNA sequences was estimated to have occurred 825 000 years ago, while the populations separated from each other 270 000 to 440 000 years ago. Additionally, the sequenced Neanderthal genome was used to detect genes potentially subjected to positive selection early in the history of our species.^4^

Later that year, Pääbo's team sequenced a draft genome from a finger bone found in Denisova Cave in southern Siberia, with coverage of 1.9x, which means that, on average, there was enough unique sequencing reads to map them to each fragment of reference genome 1.9 times. The specimen came from an archaic hominin, and the results revealed that it shared a common ancestor with Neanderthals after the divergence from anatomically modern humans. This individual, however, did not carry signs of the severe bottleneck discovered when comparing Neanderthal genomes, suggesting that it had a distinct population history. This new group of archaic hominins was named “Denisovans,” after the cave in which the first remains were discovered. This study showed the Denisovans to be less closely related to the population that contributed to the gene pool of the ancestors of modern‐day Eurasians than the Neanderthals were, stipulating that there was no gene flow into all of the Eurasians from Denisovans. On the other hand, there was a gene flow from the Denisovans into Melanesian, and roughly 4.8% of Melanesian genomes originated from Denisovans. Since Denisovans did not contribute genes to populations currently living the closest to the Altai region but did so for the Melanesian's genomes, it was argued that the Denisovans could have had a more far‐spread presence than originally anticipated.^5^

Both drafts of Neanderthal and Denisovan genomes were of low coverage, which increased the risk of the obtained sequences containing misincorporated nucleotides due to either sequencing error or DNA damage. The first draft failed to reach desirable coverage because of high contamination with exogenous DNA; the second one was characterized by shallow contamination, but the sample itself was too small, and thus the total amount of endogenous DNA was insufficient. To amend the second draft problem, Pääbo's team developed a method of preparing a single‐stranded DNA library. Thanks to this solution, in 2012, they reported the first high‐quality genome of a Denisovan. They were also the first to use a single‐stranded DNA library. The attained 30‐fold coverage allowed for direct estimation of Denisovan heterozygosity, which showed that genetic diversity among the Denisovans was surprisingly low.^6^

The application of a single‐stranded DNA library also enabled the high‐coverage genome sequencing of a Neanderthal woman from Siberia in 2014. Further studies involving other available archaic genomes and 25 modern genomes detected several gene flow events among Neanderthals, Denisovans and anatomically modern humans, thus giving evidence for possible common ancestry between hominins in the Late Pleistocene. Additionally, the team discovered that Neanderthal DNA in non‐Africans has more commonly derived alleles with Neanderthals from the Caucasus than those from Croatia or Altai mountains, suggesting that the admixture of Neanderthal genes into the modern human genome happened after the Neanderthal population split from one another. The data points toward populations of modern humans and Altai Neandertals diverging from 77 000 to 114 000 years ago, which proves that alleles common between them are a result of admixture that happened reasonably recently.^7^

Researching genomes of archaic hominins can give us insights into the demographic history of these and our species and the adaptive processes driving the changes in the genomes of every living creature. It also allows us to connect the genomic changes with the events that caused them. For example, some civilizational diseases have been proven to be a byproduct of adaptive processes. Thus investigating signals of positive selection could lead us toward variants associated with the onset of civilizational diseases. Finally, analyzing derived sequences that became fixed in anatomically modern humans after divergence from archaic hominins helps us understand their functions and identify the advantages that allowed us to prosper over our extinct relatives.

With the 2022 Noble Prize, Svante Pääbo receives recognition for his discoveries regarding genomes of extinct hominins and human evolution (Figure 1). One of the authors was pleased to participate in a plenary lecture about Neandertal Genomic given by Svante Pääbo during the XX International Congress of Genetics in 2008 in Berlin. His commitment and scientific passion impress.

**FIGURE 1:**
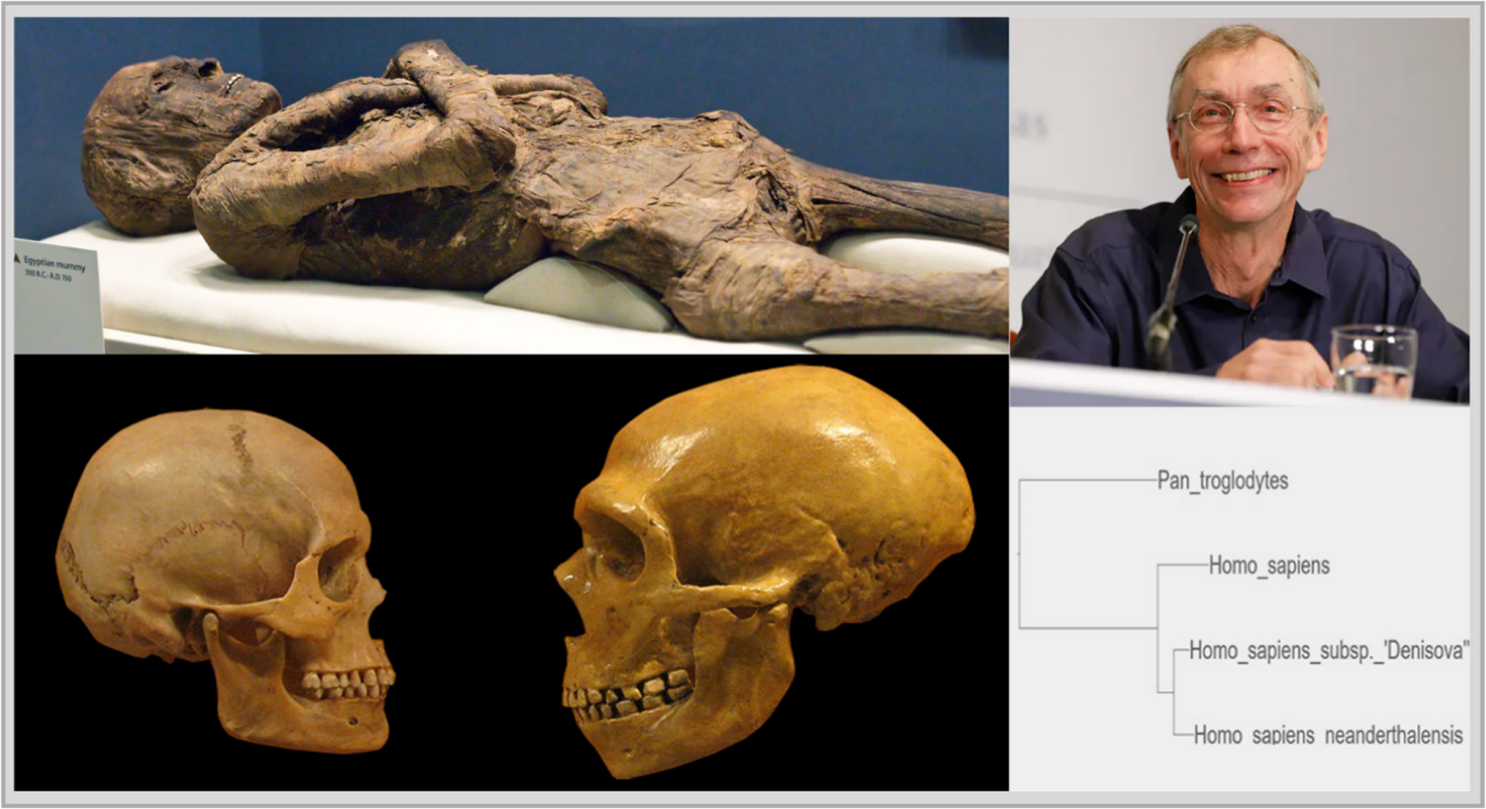
Path of Svante Pääbo's research career and the development of paleogenetics: from extracting short fragments of mtDNA from an Egyptian mummy to sequencing whole genomes of extinct hominids and discerning the evolutionary history of their and our species, for which he was awarded the Noble Prize. His first work in paleogenetics on sequences from Egyptian mummy was the world's second successful one and posed proof that nuclear DNA can survive from ancient times in a state viable for further analyses. Pääbo's further works ranged from research on sequences from other ancient samples, for example, 7000‐year‐old brain of an ancient Native American excavated from a bog in Florida to ones focusing on exploring and appraising methodologies currently available in molecular biology, for example, a publication comparing results obtained through molecular cloning and PCR. The first publication regarding genetic material from ancient hominids appeared in 1998 when Pääbo's team reported recovering mitochondrial DNA from Neanderthal. The full mitochondrial genome was sequenced in 2008, while in 2010 appeared the first draft of the Neanderthal genome, followed by high coverage Neanderthal genome in 2014. The obtained data have finally provided proof for the admixture of Neanderthal DNA to the modern human genome, which happened fairly recently (~70 000 years ago) and long after the two populations diverged (~300 000 years ago). In 2010, a new subspecies of ancient hominids was discovered—Denisovans. Results points toward them being a sister group to Neanderthals and them both sharing a latest common ancestor after the divergence from ancestors of modern humans (for greater detail, consult the remainder of the paper or the cited publications). The Figure was prepared on the bases of materials taken from: https://www.flickr.com/photos/hmnh/3033749380/; author of the original photo—hairymuseummatt; author of derivative artwork—DrMikeBaxter. Artwork licensed under Creative Commons Attribution‐Share Alike 2.0 Generic license https://creativecommons.org/licenses/by‐sa/2.0/deed.en. https://www.flickr.com/photos/126288307@N05/21860559246. Author—C Watts. Licensed under Creative Commons Attribution 2.0 Generic license https://creativecommons.org/licenses/by/2.0/. Picture of Svante Pääbo—PAP/EPA/José Luis Cereijido.

## FUNDING INFORMATION

The authors received funding from the Polish National Centre of Science, grant number 2017/26/E/NZ5/00851

## CONFLICT OF INTEREST

The authors declare no conflict of interests.
